# Ecosystem Engineer or Health Threat? Seasonal Occurrence, Farmers’ Perception and Zoonotic Parasite Load of the European Badger

**DOI:** 10.3390/ani16050770

**Published:** 2026-03-01

**Authors:** Charalampos E. Fekkas, Maria V. Alvanou, Ioannis Tsakmakidis, Katerina Melfou, Ioannis A. Giantsis

**Affiliations:** 1 Department of Agriculture, Faculty of Agricultural Sciences, University of Western Macedonia, 53100 Florina, Greece; charalamposfekkas@gmail.com (C.E.F.); gtsakmakidis@yahoo.gr (I.T.); kmelfou@uowm.gr (K.M.); igiants@auth.gr (I.A.G.); 2 Department of Animal Science, Faculty of Agriculture, Forestry and Natural Environment, Aristotle University of Thessaloniki, 54124 Thessaloniki, Greece

**Keywords:** monitoring, public health risk, parasites, *Meles meles*, agriculture pest

## Abstract

European badgers (*Meles meles*) represent an ideal model for studies on conflict resolution between pest control and conservation management. From the farmers’ perspective, badgers are considered agricultural pests, mainly owing to crop damages. From a veterinary and public health point of view, they are implicated in disease spread to livestock and humans. Nevertheless, at the same time, the European badger operates as an ecosystem engineer by creating new microhabitats. The scope of the present study is to evaluate the role, behavior, and public health importance of the European badger in an intensive agricultural area, in Galatas, western Greece. We used camera traps to collect ecological and behavioral data in correlation to seasonal occurrence of badgers. Furthermore, we collected badger feces samples to investigate any potential parasite load using both microscopic and molecular analyses. Lastly, we assessed farmers’ opinions regarding the occurrence of this animal on their properties, as well as any imputed damage. Interestingly, farmers’ opinion was in line with camera observations. Parasitological and molecular analyses revealed the presence of four parasites in badger feces, namely *Strongyloides procyonis*, *Ancylostoma caninum* and *Perostrongylus falciformis*, and a member of the family Strongyloididae. Overall, our results demonstrate the controversial role of *Meles meles* as an ecosystem engineer and a pest simultaneously, highlighting the value of effective monitoring preceding any control practice.

## Simple Summary

European badgers (*Meles meles*) represent an ideal model for studies on conflict resolution between pest control and conservation management. From the farmers’ perspective, badgers are considered agricultural pests, mainly owing to crop damages. From a veterinary and public health point of view, they are implicated in disease spread to livestock and humans. Nevertheless, at the same time, the European badger operates as an ecosystem engineer by creating new microhabitats and is protected by the Berne Convention on the Conservation of European Wildlife and Natural Habitats. The scope of the present study is to evaluate the role, behavior, and public health importance of the European badger in an intensive agricultural area, in Galatas, western Greece. We used camera traps to collect ecological and behavioral data in correlation to seasonal occurrence of badgers. Furthermore, we collected badger feces samples to investigate any potential parasite load using different techniques. Lastly, assessing farmers’ opinions regarding the occurrence of the animal on their properties and the imputed damages, we found that they were in line with camera observations. Parasitological and molecular analyses revealed the presence of four parasites and generally, our results demonstrate the controversial role of *Meles meles* as an ecosystem engineer and a pest simultaneously, highlighting the value of effective monitoring preceding any control practice.

## 1. Introduction

The European badger (*Meles meles* Linnaeus, 1758), also known as the Eurasian badger, is widely distributed throughout Europe [1]. In Greece, the species is distributed in mainland areas and has been legally protected for several decades [2]. Its population is considered stable and is classified as Least Concern (LC) by the IUCN at the European level [1]. However, information on the status and ecology of the species in Greece remains limited, and the national population is currently categorized as Not Evaluated (NE) [3]. During the period 1990–1999, increased predation on tortoise nests and a reduced frequency of juvenile tortoises were attributed to an increase in *M. meles* populations in the Alyki wetland in northern Greece [4]. In addition to mainland populations, badgers occur on the Greek islands of Rhodes and Crete, where they are classified as *Meles canescens*. Morphological comparisons of cheek teeth and analyses of mitochondrial and nuclear DNA indicate that these insular populations are conspecific with *M. canescens* from the Middle East, rather than representing an endemic subspecies (*M. m. rhodius* and *M. m. arcalus*, respectively) [5].

The European badger is a medium-sized omnivorous mammal with predominantly crepuscular and nocturnal activity patterns [6], inhabiting environments from sea level to altitudes of up to 3000 m [1]. Its diet varies seasonally and geographically, with a higher degree of carnivory observed in northern parts of its range compared to southern regions, where food resources are more diverse [7]. The diet generally includes amphibians [8], earthworms, insects, small mammals, reptiles, small birds, fruits, and seeds, particularly maize [9]. Due to its consumption of cultivated crops, especially cereals, the species may be perceived as an agricultural pest by farmers. In addition, badgers may act as scavengers [10], a behavior that has been suggested in the literature as potentially influencing disease dynamics, although this aspect remains insufficiently studied in Greece.

The European badger interacts extensively with both biotic and abiotic components of ecosystems. Through seed dispersal via feces, soil modification around burrow systems, and trophic interactions as both predator and prey, the species contributes to ecosystem processes and community structure [11]. Badger setts may also be shared with or used by other mammal species, including the raccoon dog (*Nyctereutes procyonoides*), red fox (*Vulpes vulpes*), golden jackal (*Canis aureus*), crested porcupine (*Hystrix cristata*), and Egyptian mongoose (*Herpestes ichneumon*), as well as by various smaller vertebrates and invertebrates [11].

Despite these ecological roles, the European badger has also been associated with potential negative impacts related to agriculture and public health. In rural areas, its foraging behavior often brings it into close proximity with livestock and grazing lands, particularly due to its preference for cereal crops such as maize, wheat, oats, and occasionally barley [8,9]. Such spatial overlap may increase the risk of pathogen transmission through direct contact or environmental contamination of pasture and feed [12], especially in the absence of appropriate biosecurity measures [13]. Considerable research interest has focused on the role of badgers in the epidemiology of *Mycobacterium bovis*, the causative agent of bovine tuberculosis [12]. In addition to bacterial pathogens, several parasitic taxa with zoonotic potential have been reported in European badgers, including *Eimeria* spp., *Babesia* spp., *Hepatozoon* spp., and *Leishmania infantum*, which may cause severe disease in animals and, in some cases, humans [14,15,16].

Hence, an indirect solution which can be used to prevent the transmission of diseases is the camera trap technology, used mainly for investigation of animals’ distribution range [17], species presence and animal behavior [18], as well as habitat occupancy [19] and intra-community interactions [17], which are mainly of importance for conservation management protocols development. It is also increasingly used to support conservation management, document rare behaviors, and enhance public awareness [18,19].

Within this context, the present study aims to collect ecological and behavioral information on European badgers in an intensive agricultural area of Greece using camera traps, with a focus on seasonal activity patterns and interactions between individuals. Additionally, we explore the perceived role of the species as an agricultural pest through structured questionnaires administered to local farmers. Finally, potential public health risks were evaluated through parasitological and molecular analyses of collected fecal samples, in order to assess the presence of parasites of zoonotic relevance.

## 2. Materials and Methods

### 2.1. Study Area

**Figure 1:**
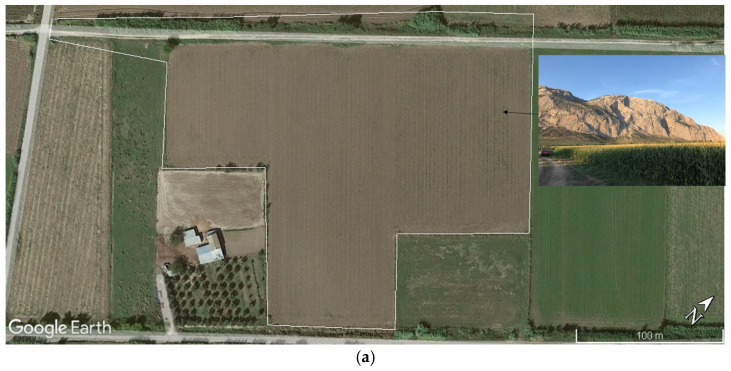

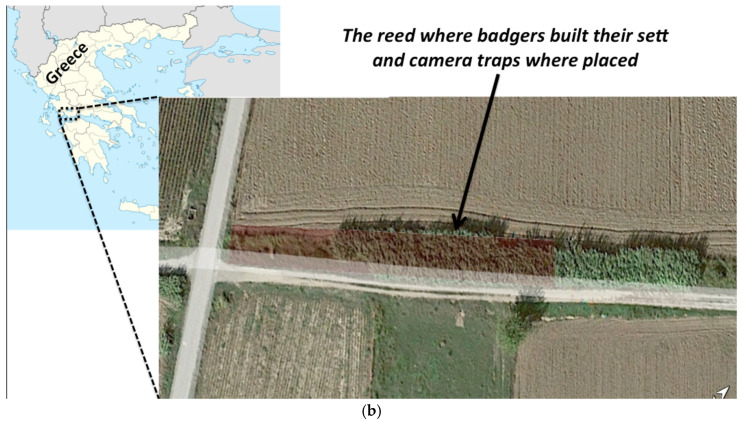
(**a**) The field of research. An aerial image of the field where research took place. Study area in Galatas, western Greece, covered by cultivated crops, where badgers were detected. (**b**) The reed where badgers built their sett and camera traps where placed.

Galatas (latitude 38°21′16.42″ N, longitude 21°34′0.58″ E) is a coastal village in the Aetolia-Acarnania Regional Unit in western Greece. It is located on the foothills of Mount Varasova in the east, with the entire area covered by arable land (Figure 1a). Due to its specific geographical location, the area of Galatas is one of the most important farming areas in the region, favoring the development of high-quality agricultural products and consequently supporting the development of many livestock farms as well. Regarding farm animals, mostly small ruminants are reared in the area. The highest percentage of cultivated crops are maize (*Zea mays*), annual clover (*Trifollium alexandrinum*), Alfa alfa (*Medicago sativa*) and olive groves (*Olea* sp.), while cotton (*Gossypium herbaceum*) and various fruit trees are found in lower percentages.

In the present study, an arable field of 3 ha (latitude 38°20′50.08″ N, longitude 21°34′31.35″ E), cultivated with maize (*Z. mays*) during the spring, was selected for investigation (Figure 1a) due to the consistent presence of European badger individuals over multiple consecutive years, their high activity levels, and their documented preference for maize seeds. As the individuals were not physically marked and could not be distinguished individually, we assumed that the total number of individuals in the population corresponded to the maximum number of badgers recorded in a single video, which was six individuals in this study. Accordingly, camera trap locations were determined based on previous field observations and information obtained through consultations with local farmers, ensuring coverage of the species’ main activity areas.

### 2.2. Camera Traps Monitoring Setup

**Figure 2:**
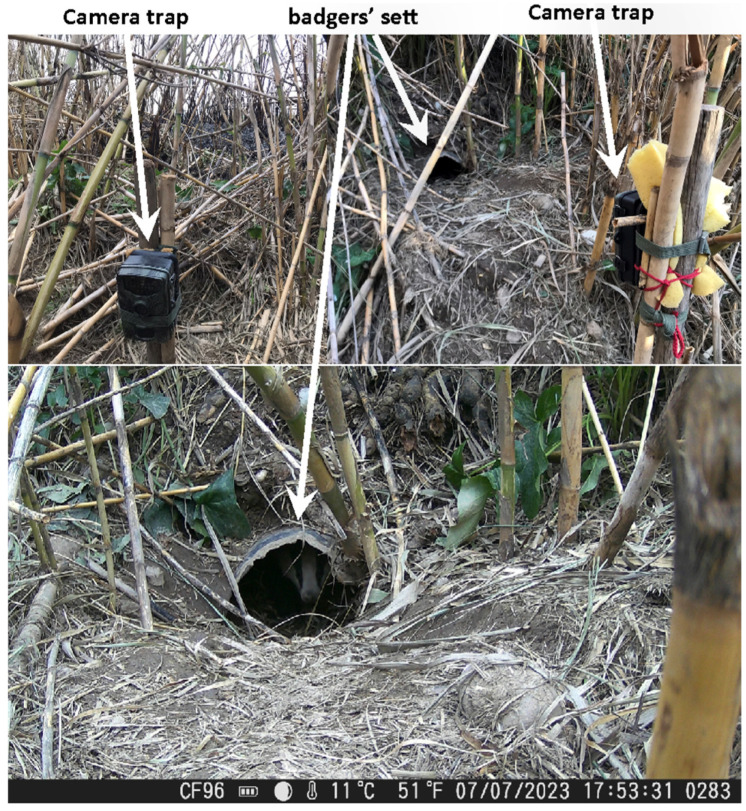
Placement of camera traps across the badgers’ sett.

We used two different camera traps for this study, The Suntek HC-900A (Shenzhen Suntek Intelligent Technology Co., Ltd., Shenzhen, China) and the HC-804A (Shenzhen Suntek Intelligent Technology Co., Ltd., Shenzhen, China). The filming period lasted one year, from June 2022 to June 2023, during which recording the behavior of the animals took place. From 27 January 2023 to 20 July 2023, camera traps were placed outside of the entrance of their sett (Figure 2), as we aimed to detect their interactions and their daily activity time. We placed the camera traps outside of the entrance of their sett based on signs of high activity such as tracks, fresh digging soil, etc. They were fixed to a wooden pole between 0.30 and 0.40 cm from the ground while facing the area of interest. Every 10 days, we visited the site to replace batteries and transfer data from SD cards to a laptop (Model:LenovoB5400, Lenovo Group Limited, Beijing, China). The camera traps were programmed to record video clips of a 30 s duration upon activation. In total, the camera traps remained in the field for 153 days. The main objective was the activity measurement throughout these months. The total duration of animals’ activity was calculated by the first and last appearance on camera. More specifically, animal activity duration was quantified by recording the time interval between the first appearance of individuals on camera and the final recorded entry into the burrow, after which no further activity was detected. Video recordings were independently analyzed by three observers, and high inter-observer consistency was achieved. Activity data were extracted through systematic and detailed review of the video footage. Because individual badgers could not be uniquely identified in the camera trap recordings and no marking technique was applied, activity estimates represent cumulative group-level activity rather than individual-specific behavioral rates. Therefore, it was not possible to determine whether the recorded activity reflects repeated movements of a few highly active individuals or contributions from multiple less active individuals. The reported activity values should thus be interpreted as an index of overall site use by the local badger group. Although camera trap monitoring was conducted between June 2022 and June 2023, quantitative analysis of activity patterns was performed only for the period January–July 2023 for which recording effort was considered sufficient and comparable among months. The remaining recordings (June–December 2022 and post-July 2023) were used primarily for qualitative behavioral observations and are not included in the statistical analysis of activity patterns. The effective sampling effort per month during the January–July period is presented in Table A1 (Appendix A). Although an effort was made to maintain comparable monitoring duration across months, complete standardization was not always feasible due to logistical constraints, camera availability, and field conditions. Therefore, minor variation in sampling effort among months should be taken into account when interpreting activity comparisons. Total monitoring days reported in Table A1 (Appendix A) correspond exclusively to the activity analysis dataset and not to the entire camera trap deployment period. While efforts were made to standardize sampling effort, complete uniformity in samples collection across months was not always achievable due to logistical constraints, variation in sample availability, and field conditions.

### 2.3. Questionnaire

To gain a better understanding of stakeholders’ perspective towards wildlife, we conducted and distributed a questionnaire consisting of mostly closed-type questions. The main goal of this questionnaire is to gain information regarding perceptions of the stakeholders on the wildlife of the region, and to evaluate their knowledge level about it. The questions aim to gather information regarding each respondent’s occupation, size of crop or farm, type of crop or farm, and income. Moreover, the questions concern wild animals’ feeding preferences, damage, and application of pest control treatment. Finally, we tried to evaluate the respondent’s opinion about whether we can consider wild animals as our allies or our enemies. The questionnaire was distributed to 86 farmers, and it was answered by 84 of them. The questionnaire survey was designed to provide complementary qualitative information to the camera trap observations. Therefore, responses were summarized descriptively to capture general trends in farmers’ perceptions and local observations. This approach was selected to document local knowledge in a region where empirical information on European badger ecology and human–wildlife interactions is limited. The questionnaire instrument is provided in Supplementary Materials to ensure transparency and reproducibility. Statistical analysis of responses was conducted applying Chi-square tests and the Pearson correlation method to explore the dependence among variables regarding farmer category and awareness of animal presence. Software IBM SPSS (version 29.0) was utilized for all analyses with significance set at *p*-value ≤ 0.05.

### 2.4. Parasitological Analyses

A total of 45 fecal samples were collected from the study area outside the setts during the whole experimental period. For the zinc sulphate method, 1 g of feces was diluted in 10 mL of tap water and passed into a centrifuge tube through a metal strainer. The tube was centrifuged at 2000 rpm for 3 min, the supernatant fluid was discarded up to 1 cm above the sediment and zinc sulfate (ZnSO_4_ 7H_2_O) 33.2% (*w*/*v*) solution was added to the sediment [20] with a few minor modifications [21]. A thorough dilution of the sediment was performed, a zinc sulfate solution was added to just above the top of the tube to form meniscus and a cover slip was placed on top. After centrifugation at 1500 rpm for 1 min, the cover slip was carefully removed and placed on a microscope slide. For the methiolate-iodine-formaldehyde-ether technique (MIF), first, 1 g of feces was diluted in a MIF solution and passed through a metal strainer into a centrifuge tube. Following that, 5 mL of ether was added into the tube, and the whole content of the tube was homogenized by intense shaking. The tube was centrifuged at 2000 rpm for 3 min. All layers of the centrifuged material, except the sediment, were discharged. Drops of the sediment were placed on a microscope slide and covered with a cover slip [20,21]. Parasitic elements were identified according to morphological characteristics under light microscopy at 400× magnification. Microscopic examination included the total prepared area [22].

Furthermore, all fecal samples were examined by a modified Ziehl–Neelsen technique for the detection of *Cryptosporidium* oocysts [23]. In brief, smears of fecal sediment were made on a microscope slide and air-dried. Following, smears were fixed transiently over a flame. Stain of smears was performed with the use of a strong carbolfuchsin solution for 5 min and then slides were heated until steam appeared [24]. After staining, the smears were washed in running water for 2 min. Slides were decolorized in acid alcohol for 30 s, counterstained with 3% methylene blue for 1 min, and rinsed and air-dried [23,25].

All parasitic stages found were identified based on morphological and morphometrical characteristics under light microscopy, at 100×, 400× and 1000× magnification [22,26].

### 2.5. Molecular Analysis

To confirm parasite and animal identification, samples were molecularly examined by PCR, targeting the conserved genomic regions of the nuclear *18S* gene and the mitochondrial cytochrome oxidase I (*COI*) gene with the primer pairs 18SF-18SR [27] and LCO1490-HCO2198 [28], respectively. DNA was extracted using the NucleoSpin DNA Stool kit (Macherey-Nagel, Düren, Germany) and concentration and purity of the DNA were evaluated using a Q5000microvolume spectrophotometer (QuawellTechnology Inc., San Jose, CA, USA). PCRs were performed in 20 μL final volume reactions, using FastGeneTaq 2x Ready Mix (Nippon Genetics, Düren, Germany) in final concentration 1X, 0.6 μL of each primer (10 mM), DNA (50 ng) and distilled water up to final volume. The PCR reaction conditions were 95 °C for 3 min, followed by 38 cycles of 94 °C, 50 °C and 72 °C for 30, 40 and 50 s, respectively, with a final step of 5 min at 72 °C. Successfully amplified products were purified with NucleoSpin Gel and PCR Clean-up Kit (Macherey-Nagel, Düren, Germany) following the manufacturer’s instructions and were bi-directionally sequenced using Sanger methodology in an ABI-Prism 3730 automatic sequencer (ThermoFisher Scientific, Waltham, MA, USA). Sequencing was conducted exclusively to confirm the taxonomic identity of the fecal samples used in the parasitological and molecular analyses. This step was necessary to ensure that all detected pathogens and parasites were unequivocally associated with *M. meles*, particularly given the potential presence of other sympatric carnivore species in the study area. The resulting phylogenetic placement of host sequences was therefore used as a methodological validation tool, and not to address population structure, phylogeography, or genetic diversity objectives. Results obtained from sequencing were used for species identification according to the percentage of similarity with previously submitted sequences in the GenBank. Quality of sequencing was performed by checking peak shape (well-defined, non-overlapping peaks), peak spacing (even spacing, consistent with expected read length), peak resolution (clear distinction between adjacent peaks) and noise (low background signal). The obtained haplotypes of the present study were edited, aligned and trimmed (at 457 and 650 base pairs for *18S* and *COI* respectively) using the software MEGA Version 7, with the MUSCLE algorithm [29]. A neighbor-joining (NJ) and a Maximum Likelihood (ML) tree were constructed for parasite *18S* and *M. meles* COI sequences, respectively, using the same software, incorporating confidence intervals obtained by 1000-replicate bootstrapping.

## 3. Results

### 3.1. Animal Activity—Behavioral Observation

**Figure 3:**
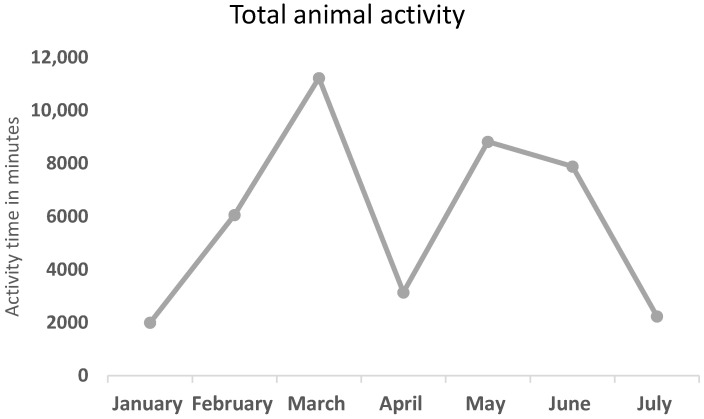
Total animal activity, counted in total min. per month, throughout a six (6)-month period.

The main goal of the camera traps application was to record if there are variations in activity patterns throughout these months. During wintertime, the activity was increased to highest levels, with a peak in March. Then, a reduction was observed between March and April, and an increase from April to May. Lastly, a reduction from summer to autumn is observed (Figure 3). More specifically, total animal activity, expressed as cumulative minutes of activity per month, showed marked temporal variation over the six-month study period from January to July. Activity levels were lowest in January (approximately 2000 min), followed by a substantial increase in February (around 6000 min), reaching a pronounced peak in March (approximately 11,000 min). In April, total activity declined sharply to about 3000 min. A second increase was observed in May, with activity rising to nearly 9000 min, followed by a slight decrease in June (approximately 8000 min). Activity levels declined again in July, reaching values comparable to those recorded in January (around 2000–2500 min). Overall, animal activity exhibited a bimodal pattern, with peaks in early spring (March) and late spring (May), and reduced activity at the beginning and end of the study period (Figure 3).

Camera trap recordings provided detailed insights into both previously described and less documented behavioral patterns of European badgers, underscoring the value of systematic behavioral observation for understanding species ecology. Below, we present the behavioral observations recorded during the study period in chronological order.

**Figure 4:**
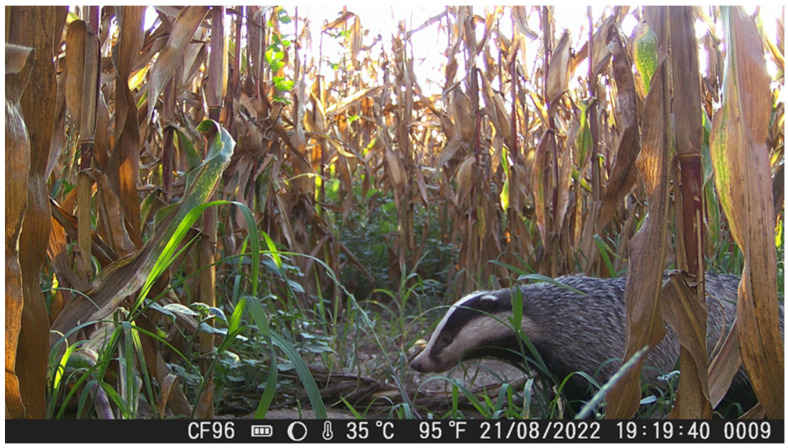
A *M. meles* individual walking through a pathway inside the maize field after trampling the maize plants during daytime.

**Figure 5:**
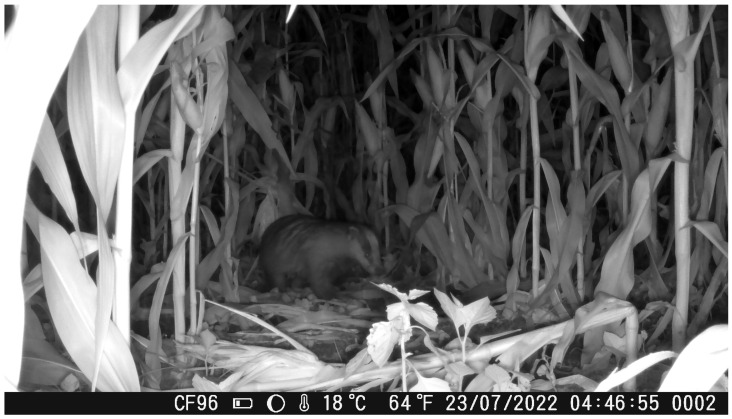
A *M. meles* individual walking through a pathway inside the maize field after trampling the maize plants during night.

During the July and August recording sessions, badgers were observed creating narrow trails within the maize fields by trampling vegetation. These trails were subsequently reused on a near-daily basis for movement within the field, suggesting an energy-efficient strategy that minimizes the need to create new pathways. Badger individuals were recorded both during night and day, whiting the maize fields (Figure 4 and Figure 5).

**Figure 6:**
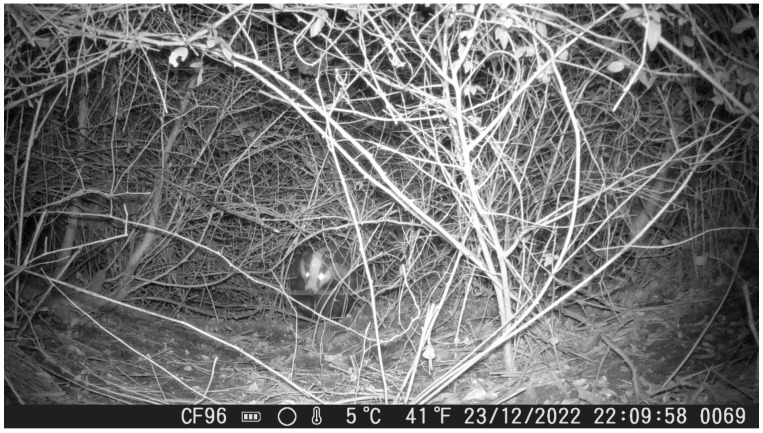
A *M. meles* individual walking through the trench which was used as a safe pathway.

At a separate recording location, a camera trap was installed outside the main sett, within the species’ home range, along a trench. The aim was to assess whether badgers used the trench as a movement corridor. Video recordings confirmed repeated use of the trench as a travel route, indicating its function as a safe and energetically favorable pathway (Figure 6).

**Figure 7:**
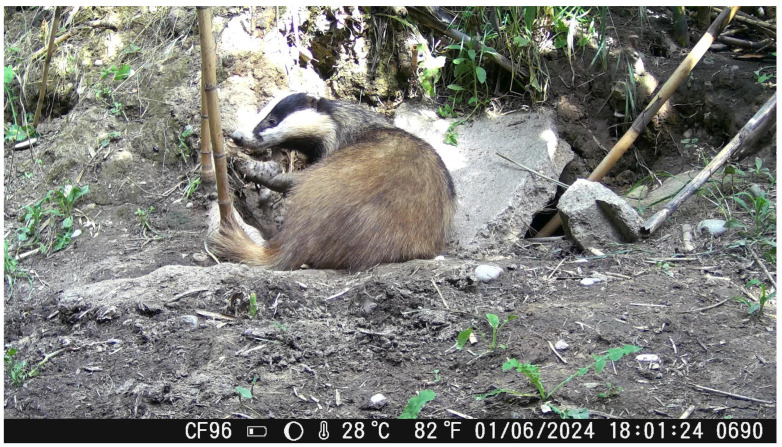
A *M. meles* individual laying down in front of the entrance of the burrow.

During January, badgers were recorded foraging for earthworms and other food resources, such as roots and plant material, during which they disturbed the soil surface, created small excavations, and damaged surrounding vegetation. In addition, although European badgers are predominantly nocturnal, daytime activity was occasionally recorded. Individuals were observed resting or lying at the entrance of their burrow during daylight hours (Figure 7).

**Figure 8:**
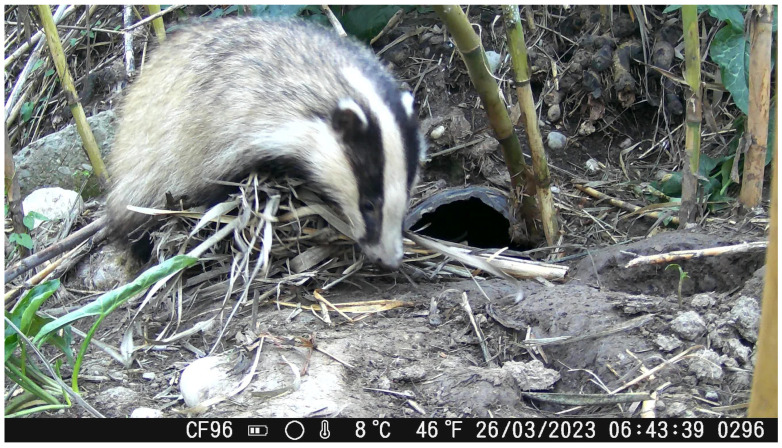
A *M. meles* individual collects substrate to transfer it in its burrow.

Disturbance near the sett was followed by a noticeable shift in emergence time on the subsequent day, with individuals exiting the burrow later than usual. Additionally, camera trap recordings documented the removal of bedding material from the sett and its deposition outside the burrow entrance. These behaviors can also be associated with ectoparasite management. As badgers frequently reuse the same resting sites, ectoparasites may accumulate in the bedding material. Individuals were observed removing bedding material from the sett and exposing it to sunlight or replacing it entirely, a behavior interpreted as bedding renewal or “airing” (Figure 8).

### 3.2. Questionnaire Results

**Figure 9:**
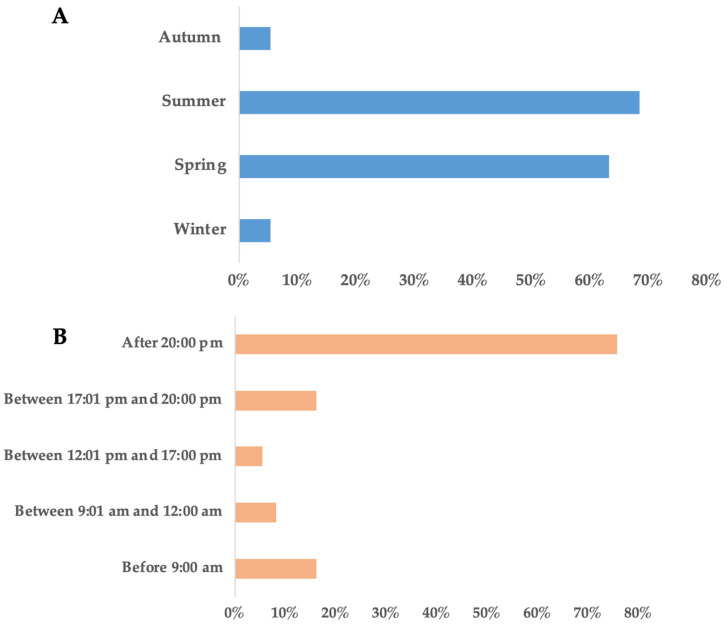
Seasonal and hourly presence of the badger according to farmers’ observations, (**A**) according to season and (**B**) according to daily hours.

Answers of participants regarding the seasonal and daily presence of the animal were in general agreement with camera recordings, as the percentage of agreement reached 63% and 68.5% during its presence in spring and summer, respectively. Concerning daily observations, most of them were made after 20:00 p.m. (75.7%), while observation between 17:00 p.m.–20:00 p.m. and before 9:00 a.m. were both 16.2% (Figure 9).

**Figure 10:**
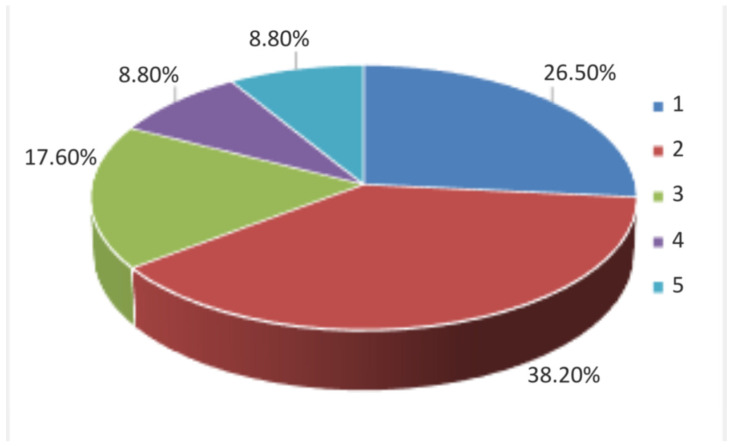
Questionnaire results indicating farmers’ opinion regarding the number of observed *M. meles* individuals.

**Figure 11:**
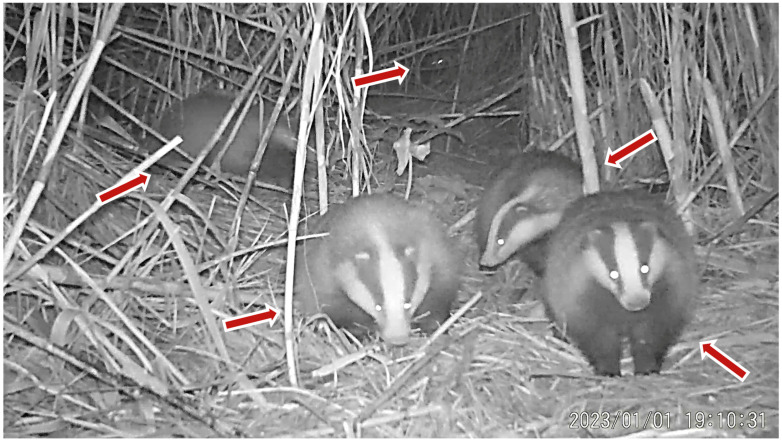
*M. meles* individuals recorded in a camera trap image depicting their social behavior. Red arrows are highlighting the different individuals.

Information obtained from the producer questionnaires complemented the camera trap data by providing an independent, perception-based perspective on European badger activity patterns. Farmers’ reports of daily activity refer to the most observations occurring during evening and nighttime hours (after 20:00 and before 09:00). This observation indicates that local producers’ perceptions accurately reflect the species’ nocturnal behavior in the study area. In addition, questionnaire data offered contextual insight into seasonal presence, perceived social interactions, and human–wildlife interactions that could not be fully captured through camera trapping alone. These observations clearly support the incidence that the European badger exhibits social behavior and most of the time acts as a nocturnal animal. Further, the vast majority of farmers mentioned that they mostly observed more than one individual, which was in accordance with camera trap results as well (Figure 10 and Figure 11).

**Figure 12:**
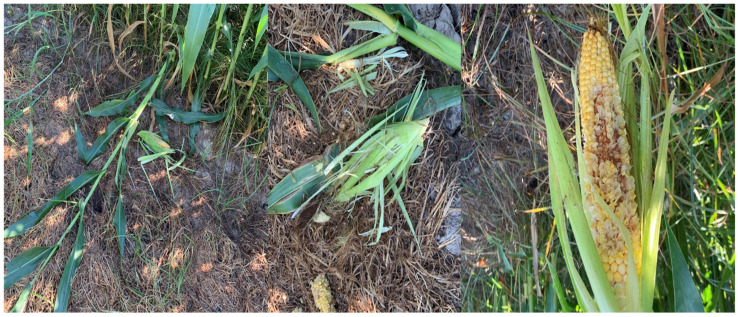
A maize plant (*Z. mays*) trampled from a badger individual. In the first picture (**left**), a destroyed plant (during the milky phase) is depicted, while in the other two, a spadix has been cut in different parts (**right**) and kernels of the corn have been removed (**middle**).

Most farmers (91.7%) reported damages on their facilities, regardless of the type of farming (crop or farm animals). According to personal observations and the answers we received from questionnaires, we observed that the damage of crops by badgers ranges between 20 and 40%. 57% of the participants answered that the damage is ~20%, while others claimed that the damage is about 40%. Accordingly, the reported crop losses represent farmers’ perceptions rather than objectively measured damage. These estimates are highly variable among farmers and are not consistently reported across the study area. In this context, crop damage was considered limited and heterogeneous at the local scale, rather than uniformly severe. Farmers considered it an agricultural pest, as was noticed on crops (Figure 12).

Furthermore, a statistically significant correlation between types of farms (crop versus animals) was observed concerning both perception of feeding preference of the European badger and pest control application (Table 1). Surprisingly, 79.8% of the responders consider *M. meles* as exclusively herbivorous, while only 19% gave the correct answer that the animal is an omnivore species (Table 1). It should be emphasized that there is a high percentage of both crop and animal farmers who apply pest control treatments throughout the year (Table 1).

Regarding control techniques, in an open-ended question, farmers reported that they apply various methods for badger-damage avoidance, which varied from use of pesticides with high toxic effects to repellents like red–white strips, or to a lesser extent more eco-friendly ones, such as scarecrows and fences. Finally, 77.3% of the participants mentioned that the badger does not contribute towards the reduction in the transmission of diseases from live, sick, and dead animals to humans.

### 3.3. Fecal Examination and Molecular Phylogeny Results

Based on microscopical screening analysis for parasites examination, we identified parasites of the families Strongyloididae, Ancylostomatidae, Filaroididae and Cryptosporidiidae. The above results were further confirmed from sequencing results. More specifically, we identified three parasites at species level, namely *Strongyloides procyonis* (Strongyloididae), *Ancylostoma caninum* (Ancylostomatidae) and *Perostrongylus falciformis* (Filaroididae), while parasites of the Cryptosporidiidae family were only identified at the family level and were submitted to the GenBank with accession numbers: PP512566, PP506743, PP507022 and PP507042 respectively. These organisms were detected after Basic Local Alignment Search Tool (BLASTn) searches of the sequenced amplified fragments on the NCBI website (https://www.ncbi.nlm.nih.gov/ (accessed on 20 February 2024)), exhibiting sequence similarities greater than 95% in comparison with congeneric haplotypes in the GenBank database. *S. procyonis* and *Cryptosporidiidae* spp. were each detected in 4 out of 45 samples, corresponding to a prevalence of 8.9% for each taxon. *A. caninum* and *P. falciformis* were detected in 3 out of 45 samples, resulting in a prevalence of 6.7% for each species. No polyspecific infestations (co-infections) were recorded in any of the analyzed samples. Parasitological results are presented as descriptive prevalence data, providing baseline epidemiological information on parasite occurrence in European badgers within the study area (Table 2).

**Figure 13:**
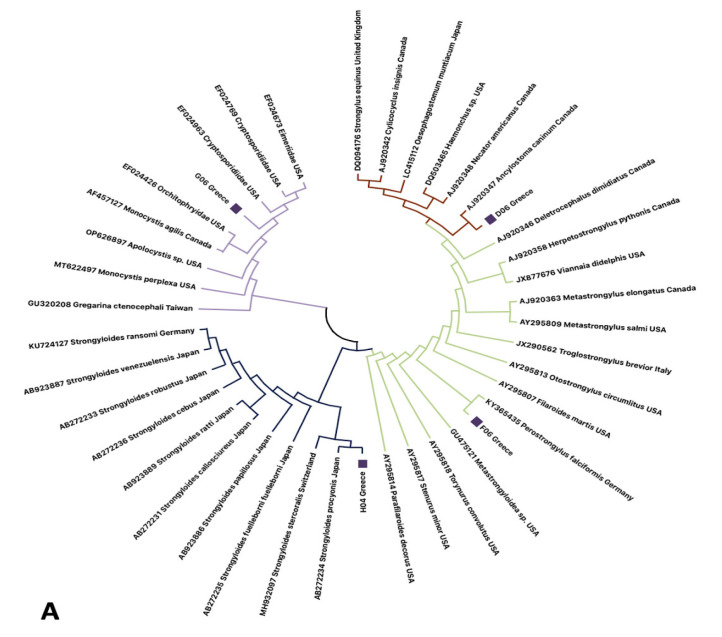

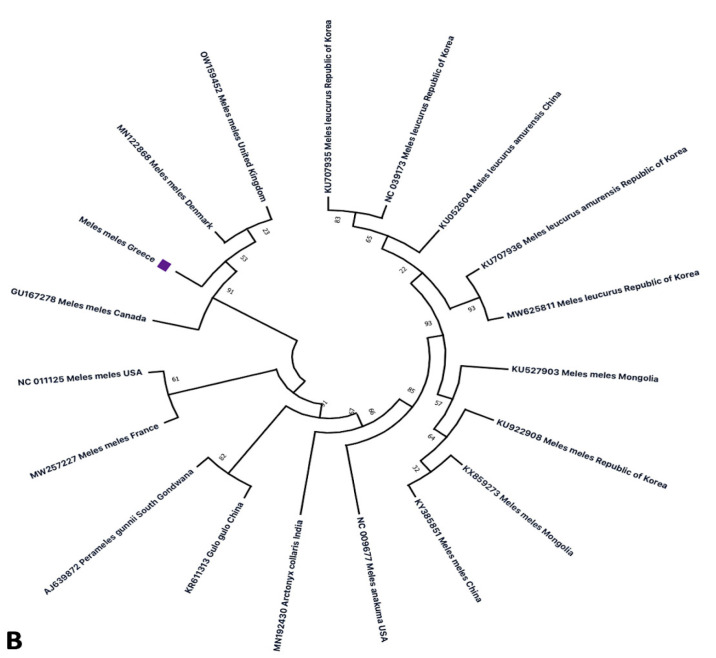
(**A**) Neighbor-joining (NJ) dendrogram depicting phylogenetic relationships of the COI haplotypes originating from M. meles feces from Greece, with the most closely related congeneric haplotypes available in the GenBank database. Accession number, taxonomic classification, and geographic origin for each haplotype obtained from the GenBank are indicated on each branch. Novel sequences derived in the present study are indicated with purple squares. Confidence intervals based on 1000 iterations are demonstrated on each clade. (**B**) Phylogenetic position of *M. meles* from Greece, in comparison with *M. meles* from various origins. Bootstrap values are indicated on the corresponding branches. Purple square indicates the novel sequence from Greece.

The sequences were phylogenetically analyzed in comparison with closely related species obtained from the GenBank database by creating a neighbor-joining (NJ) dendrogram (Figure 13A). Further, using the same methodology, we managed to identify the sequence of the European badger, as it was included in its feces. The phylogenetic analysis of *M. meles* sequences presented here serves a confirmatory purpose, verifying the species origin of the analyzed fecal samples. These results support the interpretation of the parasite and pathogen findings and are not intended as a standalone analysis of badger population genetics. *M. meles* haplotype from Greece was also submitted to the GenBank with the accession number PP506641. A Maximum Likelihood (ML) phylogenetic tree was constructed (Figure 13B).

## 4. Discussion

In the present study, useful data regarding the ecology, behavior, economic and health impact of the European badger in a specific region of agricultural importance in Greece are presented. As this species possesses many controversial roles, its monitoring is of high importance. The lack of behavioral and ecological data regarding the European badger is the first obstacle towards effective and sustainable population management of this species. Thus, this effort was a first step towards the combination of information from three different perspectives, considering *M. meles* as an agricultural pest, a disease-spread pathway, and an ecosystem engineer at the same time. Here, we investigated the behavioral and ecological pattern of the European badger combined with health and economic impacts on farmers because, according to their point of view, it is considered an agricultural pest causing serious damage to their properties.

Generally, the European badger stands as a good reason for conflict. From a farmer’s point of view, it is considered an agricultural pest. More specifically, farmers from Britain refer to badgers as an agricultural pest [30,31] mainly due to their opportunistic omnivorous nature, while a £6.5–12.5 million loss is estimated for crops in Britain annually [31]. In strict economic terms, a “pest” should only be controlled when causing an economically significant level of injury or damage, and when the costs of control are outweighed by the financial benefits accrued by that control [2].

Here in our region, according to personal observations and answers we received from the questionnaires, the damage of the badgers reaches almost half of the crop, and apart from that, they are also responsible for some damage to farmlands and infrastructure [13,32]. As ecological opportunists, they are often associated with croplands, farms, stables, granaries, food industries and many other parts of the agricultural and man-made environment [33,34,35].

However, at the same time, *M. meles*, according to Bern Convention, is categorized among protected fauna as it also operates as an ecosystem engineer. Its role mainly concerns new microhabitat establishment among its setts, which provide a suitable environment for many animal and plant species [11]. Apart from ecosystem reasons, monitoring of the emerging diseases in wildlife is also a challenging task [36]. However, gaining more knowledge of wildlife epidemiology can have beneficial effects for human and animal health, animal productivity and global biodiversity [37,38]. Plenty of infectious diseases can arise from wildlife, having a negative impact on human and animal health, significant loss on economically significant livestock species [39], and even posing a conservation threat [40].

### 4.1. Ecosystem Engineer Role Combined with Farmers’ Perspective

Farmers included in the present study possess small and medium land areas and they are characterized by relatively low income. However, their knowledge of badger behavior and ecology was insufficient. Almost all farmers that participated noticed badger individuals or badgers’ feces in their field and believed that this species is responsible for some level of damage. Apart from the damage to cultivation, ground holes and eaten roots were observed, while badgers were searching for food [2]. Furthermore, most of the participants mentioned that they apply different techniques for badger-damage avoidance, which varied from use of pesticides with high toxic effect to repellents. Farmers’ beliefs on badger diets were not adequate, with more than half of them replying that *M. meles* is exclusively an herbivore. A smaller percentage of participants answered correctly, stating that M. meles is an omnivore. Regarding feeding preferences, most of the participants answered that the animal feeds on grain, especially corn, or earthworms.

The European badger interacts with flora and fauna by dispersing plant and tree seeds and by altering the physical and chemical properties of surface soil between the burrows, shaping suitable conditions for the establishment of various plant and animal species [11]. Due to its long-term habit of creating burrows, it can cover an area of up to 970 m^2^, while the volume of excavated soil can reach up to 28 m^3^, contributing to their interaction with flora and fauna [11]. On the one hand, many farmers in Britain believe that grazing livestock are at risk of leg fractures due to badger burrowing activity in pastures and report that agricultural vehicles may also collapse into badger tunnels. Compensation claims arising from axle damage and spill-over accidents have caused some British insurance companies to refuse accident cover to farmers using vehicles or machinery in fields in which badgers have been active [2]. On the other hand, based on studies regarding piles of excavated soil by badgers, a pH increase (5.5 instead of 4.3 of other points) and higher concentrations of elements including Ca (up to three times more) and Mg (up to two times more) were observed [11]. Further, because of seed and fruit consumption, the feces deposition in their latrines, which are located near the entrance of the burrows, increases and may promote growth of vegetation [41]. Thus, they conserve a habitat full of vegetation which provides camouflage, space for their burrows and protection from enemies. Finally, almost half of the participants were not aware that the badger contributes towards reduction in the transmission of diseases from live, sick, and dead animals to humans. This is contrariwise to the accepted incidence that the badger is an opportunistic feeder, feeding on all available sources, often including carcasses of various animals, such as birds, reptiles or small mammals, thus reducing the transmission of diseases to humans and domestic animals.

### 4.2. Camera Trap Monitoring

Recordings of the present study gave us the opportunity to gain information regarding the behavior of these animals and their interaction with their habitat. Observations of early emergence and cautious behavior near the burrow entrance, particularly in May when cubs appear, suggest a potential role of vigilance and parental coordination in predator avoidance (Figure 8). Similar pre-emergence scanning behaviors have been reported in other burrowing mammals [7], indicating this may be a widespread adaptive trait. These findings highlight the importance of timing and social cues in emergence behavior, which could be further explored in relation to seasonal predation pressures and reproductive strategies. A developed sense of smell [7] is used for detection of potential enemies.

Badgers are very thorough with cleanliness, trying to get rid of different ectoparasites [42,43,44]. Construction of multiple burrows and chambers is an additional strategy to overcome ectoparasites [44]. The animals choose to sleep in a certain chamber, and when the number of parasites reaches a certain threshold, they move to alternative chambers [44]. Another advantage of multiple burrows is that they use them as a strategic plan for lower energy expenditure when they return to the closest one after grazing [45]. Additionally, after intense disturbance due to humans, livestock or domestic animals, badgers can move to other burrows for security reasons even on a daily basis [41].

Badgers utilize plant material as a nesting substrate within the sett, creating microhabitats that may support various saprotrophic invertebrates, including ticks [11]. Camera trap recordings documented periodic removal of used bedding material from the burrow entrance, followed either by exposure to ambient conditions and subsequent reuse or by replacement with fresh plant material. In some instances, individuals were observed marking the collected substrate with anal gland secretions prior to transporting it back into the sett, presumably facilitating recognition and reuse.

**Figure 14:**
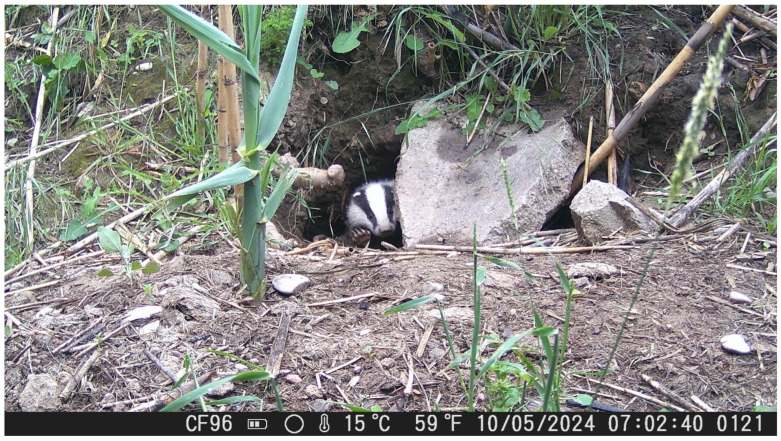
A *M. meles* puppy getting out of its burrow.

**Figure 15:**
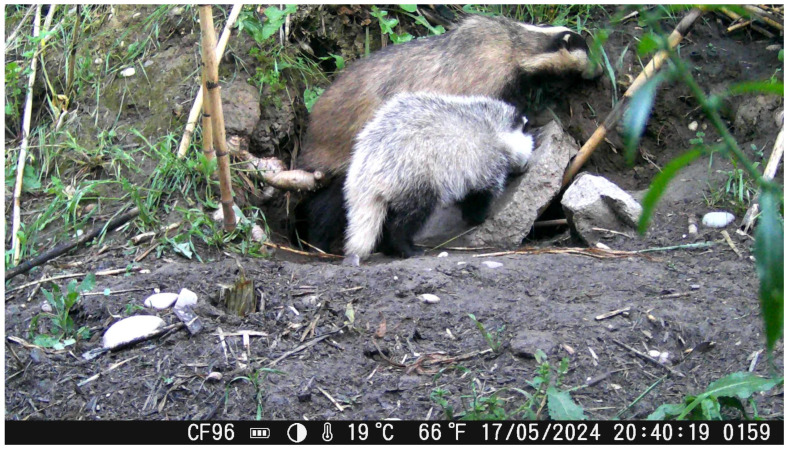
A newborn and adult *M. meles* individuals. The hair color represents a distinguishing criterion, with darker color corresponding to the adult and lighter to the newborn.

Substrate collection activity appeared to intensify during March, which may be associated with nest preparation in advance of parturition. According to the published literature, cubs open their eyes approximately one month after birth and begin emerging from the sett at 8–10 weeks of age (Figure 14 and Figure 15), while lactation may last up to approximately 2.5 months [46]. Given that the first juvenile emergence was recorded in mid-April, we infer that births in the studied population likely occurred in mid-February. This observation is consistent with previous reports indicating that most births in European badgers occur during February and March. During the monitoring period, a juvenile individual (identified based on smaller body size and color) was observed emerging from the sett for the first time (Figure 14). This observation is reported descriptively, as activity data were not systematically categorized by age class and represent cumulative group-level activity.

### 4.3. Parasite Detection

In the present study, using molecular techniques, we identified the presence of *Strongyloides. procyonis*, *Ancylostoma. caninum*, *Perostrongylus. falciformis* and Cryptosporidiidae parasites.

Apart from parasite presence, we further confirmed the animal’s (*M. meles*) presence. As it was observed from phylogenetic analyses, the present haplotype was found to be closer to the *M. meles* sequences submitted in UK and Denmark. To the best of our knowledge, this is the first *M. meles* haplotype obtained from Greece and used for DNA barcoding in Greece. Wildlife and livestock act as biological reservoirs for many parasites [47]. Among them, badgers can operate as hosts for plenty of parasites [48]. A rich in helminth parasite fauna (17 species including cestodes, trematodes and nematodes) was found in 85 European badgers sampled from Spain [49], while in a study from Ireland eight distinct helminth taxa were observed [50]. In fecal samples collected from the European badger in the UK, 14 species of protist and helminth parasites were recorded [51].

To the best of our knowledge, in the present study, the parasite *Strongyloides. procyonis* was identified for the first time in the European badger (*M. meles*), on the European and Greek level. *Strongyloides* infections were recorded in European badger populations; however, they failed to be identified at a species level [52,53,54]. It is a parasite that was first isolated in raccoons (*Procyon lotor*) in North America, while in Europe, it was initially found in raccoons in Poland and recently in Italy [55]. It has also been isolated from the Japanese badger (*Meles anakuma*) [56]. It can cause moderate dermatitis and enteritis in healthy animals (i.e., dogs); however, the effect of the infection on immunocompromised individuals [57] and endemic mesocarnivores [58] remains unexplored. Other species of the same genus were also recorded in other mustelid species in Europe (i.e., *Strongyloides martis*, *Strongyloides mustelorum*, and *Strongyloides lutrae)* [55,59,60,61].

*Perostrongylus falciformis* (Schlegel, 1933) represents a nematode parasite that was described in European badgers in Germany as *Strongylus falciformis* [62]. To the best of our knowledge, the detection of the parasite in the present study represents the first detection in Greece. *P. falciformis* is characterized by an indirect life cycle, with slugs and snails being the intermediate hosts [62]. It can be found in the European badger, and it was first isolated in Cornwall [63] and then in Italy [64]. The next records of the parasite were made in Norway [65], in Poland in 2017 [62], followed by Bosnia-Herzegovina in 2018 [66,67]. Its presence was also reported in Irish badgers (Byrne et al. 2012) and in other European countries as well [6,68].

In the present study, *Cryptosporidium* spp. members were also detected but failed to be identified at species level. It was the first identification of this parasite in Greece in the European badger. *Cryptosporidium* spp. are intracellular parasites [47] that multiply in the gastrointestinal tract of vertebrates including mammals, marsupials, birds, reptiles, amphibians and fish. In Europe, carnivorous species, including invasive ones, have been identified as carriers of parasite species of the genus *Cryptosporidium*, among them being the raccoon (*Procyon lotor*), raccoon dog (*Nyctereutes procyonoides*), fox (*Vulpes vulpes*) and the European badger as well [69]. *C. hominis* and *C. parvum* were the first recorded species of the *Cryptosporidium* genus in the European badger [70]. These organisms can affect humans [47], their food, domestic, laboratory and wild animals as well [47], especially carnivores [71].

*Ancylostomatidae* members, namely *Uncinaria stenocephala* and *Ancylostoma caninum,* were detected in wild animals in Greece (foxes and the jackal). The present study was the first time of identification of this parasite in the European badger, on the European and Greek level. Generally, *A. caninum* is characterized as quite pathogenic, with clinical symptoms ranging from diarrhea, anemia, and hypoproteinemia to even death. It is also responsible for developmental impairment [72]. Further, these parasites were detected in other European countries in high percentages in dogs, namely 33% in Romania [73] and 7.4% in Poland [74]. Apart from animals, they can have adverse impact on human health by causing enteritis, skin lesions and cutaneous larva migrans syndrome (CLM) [75,76]. The parasitological component of the present study should be interpreted within the context of certain limitations. The total number of fecal samples analyzed (n = 45) is relatively small, which may reduce the statistical robustness of prevalence estimates and limit broader epidemiological generalization. Additionally, detected prevalences were low (6.7–8.9%), and no co-infections were identified. The absence of co-parasitism may reflect sampling size constraints, temporal variation in parasite shedding, or limitations inherent to coprological detection methods. Therefore, present findings should be considered baseline descriptive data for this population, providing a foundation for future studies based on larger sample sizes and more extensive temporal monitoring.

### 4.4. Limitations

Main limitations of the study relate to sample size, monitoring methodology, and the nature of the collected data. The parasitological analysis was based on a relatively small number of fecal samples (n = 45), which may reduce the statistical robustness of prevalence estimates and limit broader epidemiological generalization. Because badgers were not individually marked, activity estimates derived from camera trap recordings represent cumulative group-level activity rather than individual behavior, making it impossible to determine whether activity reflects a few highly active individuals or several less active ones. Reported crop damage was based on farmers’ perceptions rather than objective field-based measurements, introducing potential subjectivity. Quantitative activity analysis was restricted to the January–July 2023 period, and complete standardization of sampling effort was not always feasible due to logistical constraints and camera availability. Finally, low parasite prevalences (6.7–8.9%) and the absence of co-infections may reflect limited sample size, variability in parasite shedding, or inherent constraints of coprological detection methods. Overall, the study provides baseline descriptive data that can serve as a foundation for future research involving larger sample sizes and extended temporal monitoring.

### 4.5. Highlights of the Study

The main highlights of the present study can be summarized below:

- The Dual and Contradictory Role of the European Badger: The study highlights the badger as an “ecosystem engineer” that creates new microhabitats, while simultaneously acting as an agricultural pest and a potential public health concern.
- Novel Parasitological Findings: For the first time in Greece and Europe, the parasite *S. procyonis* was detected in the European badger. Additionally, *P. falciformis* and members of the family Cryptosporidiidae were recorded for the first time in badgers in Greece. Moreover, *A. caninum* was identified in badgers for the first time at both the national (Greece) and European level.
- Activity and Behavior: Camera trap monitoring revealed that badger activity peaked in early spring (March) and late spring (May). Notable behaviors were documented, including cleaning and “airing” of nesting materials as a potential ectoparasite management strategy, as well as the use of trenches as safe transit corridors.
- Farmers’ Perception: Majority of farmers (91.7%) reported crop damage, primarily in maize fields, estimating losses between 20 and 40, a significant knowledge gap was identified, as 79.8% of respondents incorrectly believed that the badger is strictly herbivorous, whereas it is in fact omnivorous.
- Control Practices: Due to the perception of the badger as a pest species, many farmers employ control measures ranging from toxic substances to more environmentally friendly approaches such as fencing and scare devices.
- Importance of Monitoring: The study concludes that camera trap technology represents a highly effective non-invasive tool for monitoring the species and should precede any population control or management interventions.

## 5. Conclusions

In the present study, a three-perspective approach was conducted, including (a) camera trap usage, (b) questionnaire distribution to farmers, and (c) investigation of parasites in badger feces. The European badger is considered an agricultural pest, an ecosystem engineer, a threat and a barrier for disease transmission at the same time. Thus, it is a very complex issue, when it comes to management practices development for this species. In the present study, after all these months of continuous monitoring and recording of the behavior of the European badger, we observed that this animal is well adapted in many types of habitats, including human habitats, and it is familiarized with human presence. Further, farmers were not aware of its significant role in the ecosystem, which was depicted by the use of different unnecessary methods of pesticides against it. Additionally, we could claim that many times, crop damage from badgers happens accidentally, since the availability of its most important food, earthworms, is in short supply due to agricultural practices. On the other hand, wild animals are carriers of parasites, so their monitoring is of high importance. As far as the badger is concerned, it can operate as a threat to public health by transmission of some pathogens to livestock, domestic animals and to humans. Here, we identified three parasites at the species level, and another one at the family level. Camera trap technology can help for more successful monitoring of wild badger populations, as in this way we can locate them, collect samples for parasite monitoring, record its feeding preferences and damages to cultivations, while at the same time, it can contribute to the conservation of this species due to its non-invasive nature.

## Figures and Tables

**Table 1:** Respondents’ (stakeholders’) perceptions of European badger feeding behavior and pest control treatment they apply in relation to farm type.

		Crop Farmer Number (%) *	Livestock Farmer Number (%) *	Total	*p*-Value
Feeding preference	Herbivore	48 (57.1%)	19 (22.6%)	67 (79.8%)	0.004
	Carnivore	2 (2.4%)	1 (1.2%)	3 (3.6%)	
	Omnivore	4 (4.8%)	12 (14.9%)	16 (19.0%)	
Pest Control treatment	Not at all	0 (0)	21 (25%)	21 (25%)	0.002
	Seasonal	17 (20.2%)	5 (6.0%)	22 (26.2%)	
	Always	37 (44.0%)	4 (4.8%)	41 (48.8%)	

* Since some farmers both rear farm animals and cultivate crops, categorization was based on primary farming income direction.

**Table 2:** Prevalence of parasites detected in European badger (*M. meles*) fecal samples.

Parasite Species	Family	Positive Samples (n)	Prevalence (%)
*Strongyloides procyonis*	Strongyloididae	4	8.9
*Ancylostoma caninum*	Ancylostomatidae	3	6.7
*Perostrongylus falciformis*	Filaroididae	3	6.7
*Cryptosporidiidae* spp.	Cryptosporidiidae	4	8.9

## Data Availability

All data are available in the manuscript.

## Supplementary Materials

The following supporting information can be downloaded at: https://www.mdpi.com/article/10.3390/ani16050770/s1.

## Appendix A

**Table A1:** Methodology of the camera trap placement during the study. In the 1st column the coordinates of each spot where the camera trap was placed are indicated; in the 2nd column the total days of the camera trap in each spot is shown; in the 3rd column the duration of the camera trap placement is depicted; in the 4th column the purpose of each recording; and in the 5th column the number of cameras that were used is indicated.

Location (Coordinate)	Durations (Days)	Beginning	End	Purpose	Number of Cameras
38°20′48.3″ N 21°34′25.2″ E 38°20′48.3″ N 21°34′25.1″ E	65	27 January 2023	1 April 2023	Behavior	1 1
38°20′48.0″ N 21°34′24.8″ E 38°20′47.8″ N 21°34′24.6″ E	68	11 April 2023	17 June 2023	Behavior	1
38°20′47.5″ N 21°34′24.2″ E	29	22 June 2023	20 July 2023	Behavior	1
Total	175	27 January 2022	20 July 2023		
