# Supplementary material for: Ecosystem Engineer or Health Threat? Seasonal Occurrence, Farmers’ Perception and Zoonotic Parasite Load of the European Badger
Source: Animals (Basel). 2026 Mar 1;16(5):770. doi: 10.3390/ani16050770 (PMC12984882; doi:10.3390/ani16050770)
Supplement: Supplementary file 1 [file animals-16-00770-s001.zip › animals-4164546-supplementary.pdf]

**Section A. Crop Production**
**A1. Socioeconomic Characteristics**
**A1.1. What is your main occupation?**

1. Farmer
2. Livestock breeder
3. Both

**A1.2. How many stremma (1 stremma = 0.1 ha) do you cultivate?**

1. < 100
2. 100–200
3. 200–300
4. 300–400
5. 400–500
6. > 500

**A1.3. What is your annual income (€)?**

1. < 10,000
2. 10,000–20,000
3. 20,000–30,000
4. 30,000–40,000
5. > 40,000

**A2. Crop Types**
**A2.1. Which crops do you cultivate?**
*(Multiple responses allowed)*

1. Corn
2. Cereals
3. Alfalfa (*Medicago sativa*) / *Trifolium alexandrinum*
4. Olive trees
5. Citrus fruits (orange, mandarin)
6. Cotton
7. Grapevines

**A3. Crop Damage**
**A3.1. Have you ever experienced animal-related damage to your crops?**

1. Yes
2. No

If "Yes", proceed to A3.2.

**A3.2. Type of damage observed**
**Corn crops**

- Loss of seeds after sowing
- Trampling during the milky stage
- Damaged ears or leaves
- Holes in the ground

**Alfalfa / Trifolium crops**

- Holes in the ground
- Damaged or uprooted roots
- Other (specify)

**Tree crops**

- Holes in the ground
- Damaged bark

- Dead trees
- Other (specify)

**A3.3. How was the animal identified as responsible for the damage?**
1. Direct observation
2. Tracks or scats
3. Assumption
4. Similar damage observed in other fields

**A3.4. Which animal species do you believe caused the damage?**
*(Multiple responses allowed)*
- House mouse (*Mus musculus*)
- Brown hare (*Lepus europaeus*)
- Coypu (*Myocastor coypus*)
- European badger (*Meles meles*)
- Weasel (*Mustela nivalis*)
- Beech marten (*Martes foina*)
- Horse (*Equus ferus caballus*)
- Red fox (*Vulpes vulpes*)
- Golden jackal (*Canis aureus*)

**A3.5. Estimated proportion of total damage caused by each species**
1. 0–20%
2. 21–40%
3. 41–60%
4. 61–80%
5. 81–100%

**A3.6. Estimated economic cost of damage (€)**
(Open-ended response)

**A4. Control Measures**
**A4.1. Do you apply control measures against pest species?**
1. Yes
2. No

If "Yes":
**A4.2. Which species do you target?**
(Species list as above)
**A4.3. Type of control method applied**
(Open-ended response)
If "No":
**A4.4. Reason for not applying control measures**
1. Too expensive
2. Lack of knowledge or techniques
3. Ineffective
4. Environmental concerns

**A5. European Badger Observations**
**A5.1. Have you ever observed a European badger in your crops?**
1. Yes

2. No

If “Yes”:

**A5.2. Number of individuals observed**

1. 1
2. 2
3. 3
4. 4
5. ≥5

**A5.3. Season of observation**

1. Winter
2. Spring
3. Summer
4. Autumn

**A5.4. Time of observation**

1. Before 21:00
2. 21:01–00:00
3. 00:01–05:00
4. 05:01–08:00
5. After 08:00

**A6. Knowledge and Perceptions about European Badgers**

**A6.1. Self-assessed knowledge level (0–4 scale)**

0. None

1. Low
2. Moderate
3. Good
4. Very good

**A6.2. According to feeding habits, how would you classify the badger?**

1. Herbivore
2. Carnivore
3. Scavenger
4. Omnivore

**A6.3. What food items do you believe badgers consume?**

*(Multiple responses allowed)*

- Corn
- Cereals
- Fruits
- Rodents
- Sheep/Goats
- Pigs
- Cattle
- Poultry
- Reptiles
- Insects
- Earthworms

**A6.4. To what extent do you believe badgers contribute to reducing disease transmission from live, sick, or dead animals to humans?**

1. Not at all
2. Slightly
3. Moderately

4. Significantly

**Section B. Livestock Production**
*(Completed only by respondents engaged in livestock breeding)*
**B1. Livestock Characteristics**
**B1.1. Type of livestock bred**

- Sheep/Goats
- Cattle
- Pigs
- Poultry

**B1.2. Number of animals bred**
(Species-specific categorical ranges as defined in the original questionnaire)

1. Sheep – Goats
   a) 50-100
   b) 100-200
   c) <200
2. Cattle
   a) >50
   b) 50 – 100
   c) <100
3. Pigs
   a) > 50
   b) 50 – 100
   c) <100
4. Poultry
   a) >5000
   b) 5000-10000
   c) <10000

**B2. Animal Health**
**B2.1. Have you experienced disease in your herd?**

1. Yes
2. No

If "Yes":
**B2.2. Name of disease**
(Open-ended response)
**B2.3. To what extent do you believe wild animals can transmit diseases? (1–4 scale)**

1. Not at all
2. Slightly
3. Moderately
4. Significantly

**B2.4. Which wild animal species may transmit diseases?**

1. House mouse (*Mus musculus*)
2. Brown haire (*Lepus europaeus*)
3. Myocastror (*Myocastor coypus*)
4. European Badger (*Meles meles*)
5. Beech marten (*Martes foina*)

6. Weasel (*Mustela nivalis*)
7. Horse (*Equus ferus cabalus*)
8. Golden Jackal (*Canis aureus*)
9. Red fox (*Vulpes vulpes*)
10. Insects

B2.5 Do you know that wild animals can transmit diseases?

1. Yes
2. No

B.2.6 What animals species do you think that can transmit diseases in human?

1. House mouse (*Mus musculus*)
2. Brown haire (*Lepus europaeus*)
3. Myocastror (*Myocastor coypus*)
4. European Badger (*Meles meles*)
5. Beech marten (*Martes foina*)
6. Weasel (*Mustela nivalis*)
7. Horse (*Equus ferus cabalus*)
8. Golden Jackal (*Canis aureus*)
9. Red fox (*Vulpes vulpes*)
10. Insects

**B3. European Badger in Livestock Facilities**
**B3.1. Have you observed a badger in your livestock facilities?**

1. Yes
2. No

If "Yes":

- Season of observation
    1. Winter
    2. Spring
    3. Summer
    4. Autumn

- Time of observation
    1. Before 9:00 pm
    2. Between 9:01 pm – 12:00 pm
    3. Between 12:01 am – 17:00 am
    4. Between 17:01 am – 20:00 am
    5. After 20:01 am

**B4. Perceptions of Disease Transmission**
**B4.1. From 0-5 , how much do you know about badger**

0. None
1. Little
2. Averagely
3. Good
4. Very good

**B.4.2 According to feeding habits in which category would you classified the badger?**

1. Herbivore
2. Carnivore
3. Scavenger
4. Omnivore

**B4.3. What type of food do you think that a badger eat?**

1. Corn
2. Cereals
3. Fruits
4. Mice – Rats
5. Sheep – Goats
6. Pig
7. Cattle
8. Poultry
9. Reptiles
10. Insects
11. Earthworms

**B4.3 Do you think that badger induce in the transmission of diseases between animals and between animals and humans?**

1. Yes
2. No

**B.4.4 From 1-5, how much do you think that badger can induce in the reduction of transmission of diseases from live, ill, dead animals to your herd?**

1. None
2. Little
3. Averagely
4. A lot
5. I don't care

**B.4.5 From 1-5, how much do you think that badger can induce in the reduction of transmission of diseases from live, ill, dead animals to domestic animals, i.e. dog**

1. None
2. Little
3. Averagely
4. A lot
5. I don't care

**B.4.6 From 1-5, how much do you think that badger can induce in the reduction of transmission of diseases from live, ill, dead animals to humans**

1. None
2. Little
3. Averagely
4. A lot
5. I don't care
